# Predicting the outcome for patients with myelofibrosis undergoing an allogeneic hemopoietic stem cell transplant

**DOI:** 10.1038/s41408-022-00701-w

**Published:** 2022-07-26

**Authors:** Federica Sora, Sabrina Giammarco, Anna Maria Raiola, Carmen Di Grazia, Stefania Bregante, Francesca Gualandi, Riccardo Varaldo, Patrizia Chiusolo, Simona Sica, Luca Laurenti, Idanna Innocenti, Francesco Autore, Elisabetta Metafuni, Eugenio Galli, Andrea Bacigalupo, Emanuele Angelucci

**Affiliations:** 1 Dipartimento di Diagnostica per Immagini, Radioterapia Oncologica ed Ematologia, Fondazione Policlinico Universitario A. Gemelli IRCCS, Roma, Italy; 2 Sezione di Ematologia, Dipartimento di Scienze Radiologiche ed Ematologiche, Università Cattolica del Sacro Cuore, Roma, Italy; 3 UOC Ematologia e Trapianto di Midollo Osseo, IRCCS Ospedale Policlinico San Martino, Genova, Italy

**Keywords:** Health care, Medical research, Haematological diseases

**TO THE EDITOR:**

Several predictive scores have been described for patients with myelofibrosis (MF): these include IPSS [1], DIPPS [2], MYSEC [3], MIPSS [4] and GIPSS [5]. All of these have in common the identification of clinical, and biological risk factors for evolution of the disease and death. When these prognostic scores are applied to patients undergoing a hemopoietic stem cell transplant (HSCT), the outcome reflects the progression of the disease: patients with an early disease, always do better than patients with more advanced disease, and/or a higher risk score [6, 7].

The question is the following: can we identify a prognostic score, specifically designed for patients undergoing an allogeneic HSCT. A recent study has identified transplant and molecular characteristics predictive of transplant outcome, and is referred to as the MTSS or molecular and transplant scoring system. These include: age over 57, Karnovsky score < 90%, platelet counts < 150 × 10^9/L, leukocyte count > 25 × 10^9/L, an HLA mismatched donor, ASXL1 mutation, and non-CARL/MP genotype, to be independent predictors of outcome [8], The Authors incorporate these factors in 4 level MTSS, low (0–2), intermediate (3–4), high (5) and very high (>5). The OS at 5 years for these groups was 90% (low), 77% (intermediate), 50% (high) and 34% (very high). However, there is no mention of relapse in this study, and thus one wonders why introduce molecular prognostication.

In our own series of patients, we asked the question whether the same prognostic value could be achieved with less information as compared to the MTSS. We have previously reported that spleen size, (maximum spleen longitudinal size recorded on ultrasound pretransplant—with a cut off of 22 cm), and transfusion burden (with a cut off of 20 red blood cell transfusions) were predictive of outcome after HSCT [9]. A recent study in patients with myelofibrosis grafted from haploidentical donor, has confirmed a negative impact of a large spleen (>22 cm) on relapse, but not on survival [10], and splenectomy may be beneficial in patients with a very large spleen [11]. The role of transfusion burden has not been evaluated to our knowledge.

We therefore analyzed 157 patients with myelofibrosis undergoing an allogeneic HSCT at a median interval of 925 days from diagnosis (116–8865). The clinical characteristics were as follows: 110 and 47 patients were aged < =/>60 years; 71 were prepared with a conditioning regimen including fludarabine and one alkylating agent (1 alk) (busulfan, melphalan or thiotepa) [12], and 86 patients received a regimen with two alkylating agents (thiotepa busulfan and fludarabine) (TBF) [12].

Fifty two patients exhibited a maximum spleen size before HSCT of ≤ 22 cm and had received 0–20 red blood cell transfusion pre HSCT (TS = 0), whereas 105 patients had a large spleen (>22 cm) and/or a heavy transfusion burden (>20 transfusions) (TS = 1). Spleen size was recorded as maximum size, also if the patient was splenectomized pre-transplant. Patients were also stratified according to the dynamic international prognostic scoring system (DIPSS) as int1-int2 (*n* = 87) or high risk (*n* = 70). Finally 60 patients were grafted from HLA identical sibling and 97 from matched unrelated or mismatched related donors. We looked at three outcomes: disease free survival (DFS) the event being death or relapse; transplant related mortality (TRM) the event being death without relapse; and relapse, the event being relapse of myelofibrosis.The multivariate Cox analysis included TS, age >60 years, DIPSS score, conditioning regimen and donor type.

The 5 year DFS was 51% vs 42% (*p* = 0.09) for patients aged < =60/> years, it was 60% vs 36% (*p* = 0.002) for patients prepared or not with TBF, and 59% vs 34% (*p* = 0.002) for patients with int1-int2 or high DIPSS. When looking at TS the 5 year DFS was 74% vs 36% (*p* = 0.0001) for patients with low or high TS (Fig. 1). The 5 years DFS for patients grafted from identical or alternative donors was 53% vs 46% (*p* = 0.3).

**Fig. 1:**
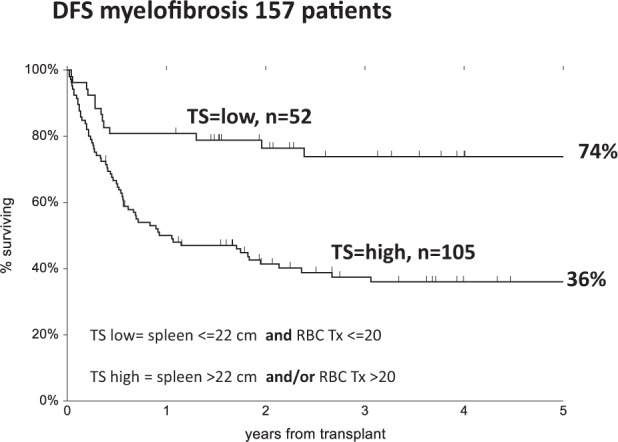
Disease free survival of 157 patients with myelofibrosis undergoing an allogeneic hemopoietic stem cell transplant. A low transplant score (TS) is identified as red blood cell transfusions (RBC Tx) <20 units and spleen size less than 22 cm. A high TS is identified as red blood cell transfusions (RBC Tx) >20 units and/or spleen size than >22 cm.

In multivariate Cox analysis (Table 1) patients age predicted only TRM (HR 1.8, *p* = 0.02); the conditioning regimen had a strong impact on relapse (HR 0.1, *p* < 0.00001) and therefore DFS (HR 0.3, *p* < 0.0001); TS had a significant impact on all three outcomes: DFS (HR 2.0, *p* = 0.008); TRM (HR 2.1, *p* = 0.03); Relapse (HR 2.2, *p* = 0.04). DIPSS predicted DFS, whereas donor type was the least predictive variable (Table 1).

**Table 1:** Cox analysis on DFS, TRM, relapse.

DFS				*P*	TRM HR (95%CI)	*P*	REL HR (95%CI)	*P*
Var	Base	Comp HR (95%CI)						
Age	≤60	>60 yy	1.4 (0.8–2.1)	0.1	1.8 (1.0–3.3)	0.02	0.8 (0.4–1.9)	0.7
Cond	1 alk	TBF	0.3 (0.2–0.5)	0.0000	0.6 (0.2–1.2)	0.1	0.1 (0.4–0.2)	0.0000
TS	0	1–2	2.0 (1.2–3.4)	0.008	2.1 (1.0–4.3)	0.03	2.2 (1.0–5.1)	0.04
DIPSS	in1-2	high	1.6 (1.0–2.6)	0.02	1.5 (0.8–2.8)	0.1	1.8 (0.9–3.8)	0.09
Donor	sibs	other	1.6 (1.0–2.7)	0.04	1.8 (0.9–3.7)	0.08	1.3 (0.6–2.8)	0.3

*DFS* disease free survival, *TRM* transplant related mortality, *REL* relapse, *P P*-value, Var variable, *Bas* baseline value, *Comp* compared value, *HR* hazard ratio, 95%*CI* 95% confidence interval, yy years, *Cond* conditioning, 1alk one alkylating agent, *TBF* thiotepa, busulfan, fludarabine, *DIPSS* Dynamic International Prognostic Scoring System, *int1* intermediate 1, Donor stem cell donor type, *Sibs* matched siblins, *Other* other donor type.

Causes of death in patients with a high TS as compared with patient with a low TS were as follows: infectious deaths were 13% vs 5%, multiorgan toxicity 11% vs 4%, graft failure 4% vs 2%, GvHD 3% vs 4%, other transplant related 5% vs 6%, and relapse 25% vs 8%. When looking at patients over 60 years of age, prepared with two alkylating agents (TBF) (*n* = 33), the 5 years DFS was 79% for low TS vs 26% for a high TS (*p* = 0.007). Therefore, it seems possible to achieve excellent DFS also in older patients, given that they come to transplant without a heavy transfusion burden or a spleen that occupies the whole left abdomen. The problem remains for the older patients, over 60 years, with a high TS, in whom the TRM reaches 50%: one may have to reduce the intensity of the conditioning regimen in such patients.

As shown in the multivariate Cox analysis, TS predicts both transplant related death and relapse, possibly because spleen size and transfusion burden are surrogates for advanced disease and/or time from diagnosis, and at the same time, have implications for transplant events: a large spleen can delay hematologic recovery and increase infectious complications and a high transfusion burden generates sensitization to HLA antigens, and may increase the risk of graft failure.

The drawback of the study is of course its retrospective nature, but on the other side the number of patients involved is relatively large.

In conclusion we confirm that maximum spleen size and pre-HSCT transfusion burden are strong predictors of outcome for patients with myelofibrosis, and predict both transplant related toxicity as well as relapse of the original disease. Patients with a high TS may be eligible for programs of reduced toxicity conditioning regimens.

## Data Availability

The data of the analysis on which this report is based, is available on request.
